# Assessment of Three Tropospheric Delay Models (IGGtrop, EGNOS and UNB3m) Based on Precise Point Positioning in the Chinese Region

**DOI:** 10.3390/s16010122

**Published:** 2016-01-20

**Authors:** Hongxing Zhang, Yunbin Yuan, Wei Li, Ying Li, Yanju Chai

**Affiliations:** 1 State Key Laboratory of Geodesy and Earth’s Dynamics, Institute of Geodesy and Geophysics, No. 340 Xudong Road, Wuhan 430077, China; yybgps@whigg.ac.cn (Y.Y.); liwei@whigg.ac.cn (W.L.); liying@whigg.ac.cn (Y.L.); cyjigg@whigg.ac.cn (Y.C.); 2 University of Chinese Academy of Sciences, No. 19A Yuquan Road, Beijing 100049, China

**Keywords:** zenith tropospheric delay, IGGtrop model, EGNOS model, UNB3m model, precise point positioning

## Abstract

Tropospheric delays are one of the main sources of errors in the Global Navigation Satellite System (GNSS). They are usually corrected by using tropospheric delay models, which makes the accuracy of the models rather critical for accurate positioning. To provide references for suitable models to be chosen for GNSS users in China, we conduct herein a comprehensive study of the performances of the IGGtrop, EGNOS and UNB3m models in China. Firstly, we assess the models using 5 years’ Global Positioning System (GPS) derived Zenith Tropospheric Delay (ZTD) series from 25 stations of the Crustal Movement Observation Network of China (CMONOC). Then we study the effects of the models on satellite positioning by using various Precise Point Positioning (PPP) cases with different tropospheric delay resolutions, the observation data processed in PPP is from 21 base stations of CMONOC for a whole year of 2012. The results show that: (1) the Root Mean Square (RMS) of the IGGtrop model is about 4.4 cm, which improves the accuracy of ZTD estimations by about 24% for EGNOS and 19% for UNB3m; (2) The positioning error in the vertical component of the PPP solution obtained by using the IGGtrop model is about 15.0 cm, which is about 30% and 21% smaller than those of the EGNOS and UNB3m models, respectively. In summary, the IGGtrop model achieves the best performance among the three models in the Chinese region.

## 1. Introduction

Tropospheric delays are some of the most significant error sources in the data analysis of the space geodetic observations like satellite navigation. Tropospheric delays consist of hydrostatic (dry) and water vapor (wet) parts. Many mitigation approaches have been developed for various user groups. The dry portion of the ZTD can be accurately modeled with the observed surface pressure, while estimation of the wet portion of ZTD is a standard method in most post high-accuracy geodetic applications for the highly variability of the wet portion [1]. However, some navigation tasks like real-time Positioning, Navigation and Timing (PNT) applications require users to process the data in real-time mode, so choosing a more accurate empirical model to mitigate the tropospheric delay will be necessary and crucial for real-time PNT users [2,3,4,5].

The commonly used empirical tropospheric delay models include: the UNB models (UNB1 through UNB4) and the EGNOS model [6,7,8,9,10], which is the WAAS version of UNB3m, *etc.* However, as latitude-only based models, the UNB3m and EGNOS models often cause large biases in some areas where ZTD deviate a lot from the zonal average [4]. In recent years, lots of efforts have been made to develop multi-dimensional grid tropospheric delay models for regional or global applications, such as the GPT model and its updated versions GPT2 and GPT2w, the TropGrid model with its updated version TropGrid2, and the 3D grid-based IGGtrop model [4,5,11,12,13,14,15].

The IGGtrop model is initially developed to provide tropospheric delay for the users of Chinese BeiDou Navigation Satellite System (BDS) and the area augmentation system based on BDS in China. It uses a 3D grid to calculate ZTD to obtain more homogenous performances for different areas of China and it can also be applied to a global scale with relatively good performance [4]. In order to optimize the storage of the parameters for the IGGtrop model and make it more convenient in applications, Li *et al.* developed the new versions of IGGtrop called IGGtrop_*r_i_* (*i* = 1, 2, 3) [5]. 

Many works have been done to evaluate the performances of the tropospheric delay models [4,5,16,17,18,19,20,21,22,23,24]. However, the data used and the time span for the evaluations for Chinese region are not sufficient, especially for the IGGtrop model. In addition, the evaluations for the tropospheric delay models are mostly based on the comparisons of the ZTDs calculated by empirical models (referred to as model-derived ZTD) and the ZTDs derived from GPS observations (referred to as GPS-derived ZTD) or other technical measures, currently. However, this is not sufficient since the references used in the evaluations are not really “true” values and it is also important to know the performances of different tropospheric delay models in satellite positioning. 

In this paper, aiming at providing references for the selection of empirical tropospheric delay models for GNSS users in Chinese region, we comprehensively evaluate three main empirical tropospheric delay models, *i.e.*, the IGGtrop (the original version), EGNOS and UNB3m models, under typical Chinese atmospheric conditions. An integrated evaluation scheme consisted of two major steps is designed. In the first step, the accuracies of empirical models are assessed by comparing the model-derived ZTDs with 5 years’ GPS-derived ZTDs at 25 stations of CMONOC covering different latitudinal bands of China. The variations of the models’ errors are analyzed in both spatial and temporal domains. In the second step, we implement the tropospheric delay models in PPP to process a whole year’s GPS data from 21 stations of CMONOC. The accuracies of the PPP solutions would reveal the performances of the IGGtrop, EGNOS and UNB3m models in satellite positioning.

This paper is arranged as follows: Section 2 introduces the methodology and the data we used for the assessment. Section 3 presents the results of comparisons for ZTDs obtained from different models and also their effects on satellite positioning. The conclusions are given in Section 4. 

## 2. Methodology and Data

### 2.1. Assessment of Tropospheric Delay Models Based on GPS-Derived ZTDs

The method to assess the tropospheric delay models in this step of evaluation is to compare model-derived ZTDs with GPS-derived ZTDs. The accuracies of the IGGtrop, EGNOS and UNB3m models are assessed using four quantities *i.e.*, the Bias, RMS, Standard Deviation (STD) and relative errors $I_{\text{rel}}$. The equations for the calculation of Bias, RMS, STD and $I_{\text{rel}}$ are as follows:

(1) $$\text{Bias}=\frac{1}{m}\overset{m}{\underset{i=1}{\sum }}\left({\text{ZTD}}_{i}^{\text{model}}-{\text{ZTD}}_{i}^{\text{GPS}}\right)$$

(2) $$\text{RMS}=\sqrt{\frac{1}{m}\overset{m}{\underset{i=1}{\sum }}{\left({\text{ZTD}}_{i}^{\text{model}}-{\text{ZTD}}_{i}^{\text{GPS}}\right)}^{2}}$$

(3) $$\text{STD}=\sqrt{\frac{1}{m}\overset{m}{\underset{i=1}{\sum }}{\left({\text{ZTD}}_{i}^{\text{model}}-{\text{ZTD}}_{i}^{\text{GPS}}-{\text{Bias}}^{\text{model}}\right)}^{2}}$$

(4) $$I_{\text{rel}}=\frac{\text{RMS}}{\frac{1}{m}\sum_{i=1}^{m}\left({\text{ZTD}}_{i}^{\text{GPS}}\right)}$$

where: ${\text{ZTD}}_{i}^{\text{GPS}}$ is GPS-derived ZTD; ${\text{ZTD}}_{i}^{\text{model}}$ is model-derived ZTD; ${\text{Bias}}^{\text{model}}$ is the bias value of the corresponding model calculated by Equation (1); m is the number of the data points of ZTD. 

**Figure 1:**
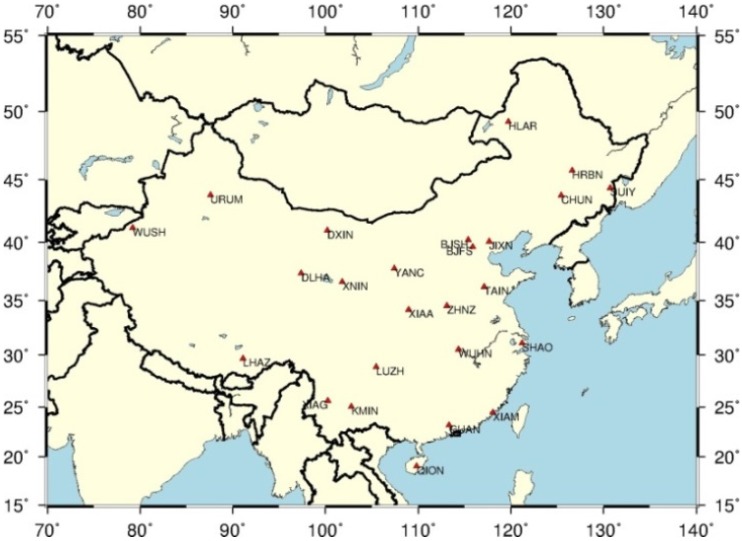
Distribution of 25 selected stations of CMONOC used in this study.

A uniformly distributed GPS data set (1.1.2009–31.12.2013) collected at 25 stations from CMONOC is used to estimate the GPS-derived ZTDs. The GPS-derived ZTDs with a temporal resolution of 2-h are estimated by ionosphere-free-based PPP algorithm using International GNSS Service (IGS) final orbits and clocks. The distribution of the 25 stations is shown in Figure 1.

In order to illustrate the accuracy of the GPS-derived ZTDs estimated by the PPP in this paper, we compare the GPS-derived ZTDs with the corresponding IGS final ZTD products. The accuracy of the IGS final ZTD product is specified with 4 mm by the IGS Central Bureau. The GPS data is from six IGS stations in China, *i.e.*, BJFS, XIAN, LHAZ, SHAO, WUHN and URUM, over one-week period. The results show that the differences between the GPS-derived ZTDs and IGS final ZTD products vary from −4 mm to 13 mm with the mean value of about 0.5 mm and mean RMS of about 4.9 mm, and this indicates a comparable accuracy with the IGS final ZTD product. Therefore, we believe that the GPS-derived ZTDs calculated by the PPP in this paper can be regarded as references for the valuations of empirical tropospheric delay models.

### 2.2. Assessment of Tropospheric Delay Models Based on PPP 

Conventional PPP algorithm is a technique that uses precise orbit, clock and ionosphere-free observations for highly accurate positioning to achieve centimeter or decimeter level accuracy. The zenith tropospheric delay is estimated along with the position and ambiguity parameters in the conventional PPP algorithm [25,26,27,28,29]. In this step of evaluation, the conventional PPP algorithm is modified according to different tropospheric delay resolutions: using different empirical models (*i.e.*, IGGtrop, EGNOS, UNB3m) with Niell mapping function (NMF) [30] to correct the tropospheric delay directly rather than estimate it during processing. We apply the conventional and modified PPP into this step of evaluation. In addition, the network solution from the GAMIT-GLOBK (version 10.5) software package with an accuracy of millimeter is used as the reference to evaluate the accuracies of the PPP solutions. The selected time interval for the network solution is 10-day throughout the whole year of 2012 which is considered to be sufficient for our purpose since the coordinates of the stations show little variations in short term. The positioning errors of the PPP solutions are analyzed in both spatial and temporal domains.

All PPP solutions here use the IGS final orbits and clocks, 15-degree elevation cutoff angle. The errorsof a daily solution for PPP in vertical and horizontal (${\text{Error}}_{\text{vertical}}$, ${\text{Error}}_{\text{horizontal}}$) components are calculated as:

(5) $${\text{Error}}_{\text{vertical}}=\frac{1}{m}\overset{m}{\underset{i=1}{\sum }}\left|u_{i}-u_{r}\right|$$

(6) $${\text{Error}}_{\text{horizontal}}=\frac{1}{m}\overset{m}{\underset{i=1}{\sum }}\sqrt{{\left(n_{i}-n_{r}\right)}^{2}+{\left(e_{i}-e_{r}\right)}^{2}}$$

where: $n_{i},\;e_{i}$ and $u_{i}$ are the coordinates estimated by PPP in north, east and up components at epoch i, respectively; $n_{r},e_{r}$ and $u_{r}$ are the coordinates obtained from the network solution; m is the number of samples after PPP reached convergence. The criterion for determining whether the solution reaches convergence is the formal error (δ < 2 cm) of the position parameter.

The data used in this step of evaluation includes one year’s (1.1.2012–31.12.2012) GPS data from 21 nationally distributed stations of CMONOC. This set of 21 well-performing stations represents high, middle and low latitudinal regions of China and is considered to be a sufficient data sample for our purpose. In view of the data availability of CMONOC and the negligible frequency dependence of the tropospheric path delay for navigation signal [1], the analysis given in this step of evaluation is based on GPS data and the results can also provide references for other GNSS users in Chinese region.

## 3. Results 

### 3.1. Accuracy Analysis for the IGGtrop, EGNOS and UNB3m Models Against GPS-Derived ZTDs

We listed the mean bias, STD, RMS and relative errors of the ZTDs calculated by the IGGtrop, EGNOS and UNB3m models at 25 stations in Table 1. The STD values are at a level of 5.1 cm for both the EGNOS model and the UNB3m model, and decrease to 4.3 cm when utilizing IGGtrop. The RMS of the IGGtrop model is 4.4 cm, which is about 24% and 19% smaller than that of the EGNOS model and the UNB3m model, respectively. Furthermore the biases of IGGtrop vary from −1.0 cm to 1.0 cm at most of the stations (19 of the 25 stations). However, for the EGNOS and UNB3m models, the biases exceed 1.0 cm at most of the stations (22 of the 25 for EGNOS and 19 of the 25 for UNB3m). This indicates that the ZTDs provided by the EGNOS and UNB3m models have systematic biases in China. The biases of IGGtrop exceed 3.0 cm at 2 stations (QION and GUAN) locating at low latitudinal regions indicating a relatively low accuracy for the IGGtrop model at low latitudes. These findings are in agreement with those found by Li *et al.* [4,5]. The relative errors of IGGtrop are smaller than 3.0% at all stations. However, the relative errors exceed 3.0% at 8 stations for EGNOS and 5 stations for UNB3m. In addition, the STD, RMS and relative errors of IGGtrop are smaller than those of EGNOS and UNB3m at almost all stations. The above results suggest that the IGGtrop model can provide more accurate estimations of ZTD than the EGNOS and UNB3m models in China.

**Figure 2:**
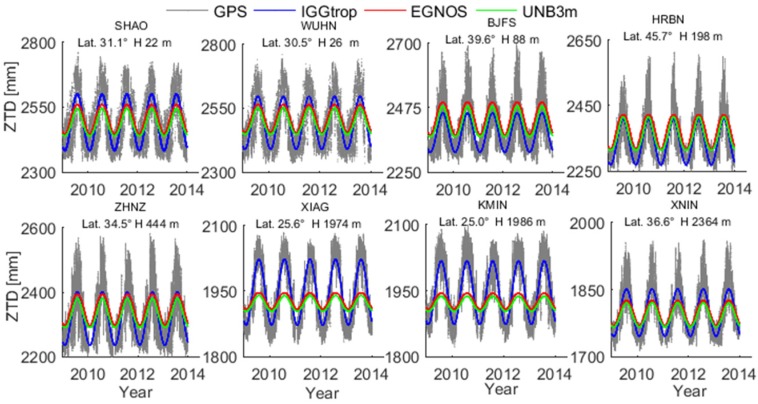
Time series of ZTD derived from GPS and three empirical models (IGGtrop, EGNOS and UNB3m) over the time period from 2009 to 2013.

Figure 2 illustrates the GPS-derived ZTDs and the corresponding ZTDs calculated by the IGGtrop, EGNOS and UNB3m models at eight exemplary stations over five-year period from 2009 to 2013. The selected eight stations locate at various altitudinal regions from low to high in China, and we therefore can examine the variations of model-derived ZTDs with respect to altitude. It can be seen that the ZTDs calculated by IGGtrop are much closer to the GPS-derived ZTDs than those of the EGNOS and UNB3m models at eight selected stations.

**Table 1:** Statistics of ZTD estimation errors (Bias, STD, RMS and relative errors) for the IGGtrop, EGNOS and UNB3m models at 25 stations in China during the period 2009–2013.

SITE		IGGtrop				EGNOS				UNB3m			
	H	Bias	STD	RMS	Rel	Bias	STD	RMS	Rel	Bias	STD	RMS	Rel
	(m)	(cm)	(cm)	(cm)		(cm)	(cm)	(cm)		(cm)	(cm)	(cm)	
SHAO	22	0.1	6.7	6.7	2.7%	1.5	7.7	7.8	3.1%	−0.4	7.6	7.6	3.0%
WUHN	26	0.0	6.2	6.3	2.5%	1.9	7.3	7.6	3.0%	0.3	7.3	7.3	2.9%
GUAN	31	−3.0	6.2	6.9	2.7%	−2.5	7.3	7.8	3.0%	−4.8	7.7	8.0	3.1%
JIXN	39	−1.3	5.1	5.3	2.2%	4.3	5.5	7.1	2.9%	2.3	5.3	5.8	2.4%
BJFS	88	−0.9	5.3	5.4	2.3%	4.4	5.7	7.2	3.0%	2.4	5.5	6.1	2.5%
XIAM	106	−1.3	5.9	5.9	2.3%	−1.4	6.7	6.8	2.7%	−3.5	6.9	7.7	3.1%
BJSH	155	−0.9	5.2	5.3	2.2%	3.8	5.6	6.8	2.9%	1.8	5.4	5.7	2.4%
HRBN	198	−0.4	4.0	4.0	1.7%	3.1	4.4	5.5	2.4%	1.5	4.1	4.4	1.9%
QION	208	−3.4	4.8	5.8	2.3%	−1.9	6.1	6.3	2.5%	−3.7	6.4	7.3	2.9%
CHUN	268	−2.3	4.3	5.0	2.1%	2.1	4.8	5.3	2.3%	0.4	4.5	4.6	2.0%
LUZH	298	2.3	4.3	4.9	2.0%	−0.9	6.0	6.1	2.5%	−2.5	6.1	6.6	2.7%
TAIN	339	−0.5	5.5	5.5	2.4%	3.1	6.2	7.0	3.0%	1.2	6.1	6.3	2.7%
SUIY	369	−0.9	3.8	3.8	1.7%	2.7	4.4	5.2	2.2%	1.1	4.2	4.3	1.9%
ZHNZ	444	0.0	5.5	5.5	2.4%	2.9	6.3	7.1	3.1%	1.2	6.3	6.4	2.8%
XIAA	509	−0.2	5.1	5.2	2.3%	2.1	6.1	6.6	2.9%	0.4	6.1	6.2	2.7%
HLAR	629	0.0	3.3	3.3	1.5%	2.2	3.7	4.3	2.0%	1.0	3.3	3.5	1.6%
URUM	859	0.8	2.9	3.0	1.4%	2.4	2.8	3.8	1.8%	1.2	2.7	3.0	1.4%
DXIN	1018	1.0	2.9	3.1	1.5%	4.3	3.1	5.3	2.5%	3.1	2.9	4.2	2.0%
YANC	1304	0.9	3.9	4.0	2.0%	2.4	4.3	5.0	2.4%	1.2	4.3	4.5	2.2%
WUSH	1395	0.7	2.7	2.8	1.4%	1.6	2.6	3.1	1.5%	0.6	2.5	2.6	1.3%
XIAG	1974	0.3	2.8	2.8	1.4%	−1.7	5.8	6.0	3.1%	−2.4	5.8	6.3	3.2%
KMIN	1986	−0.7	3.0	3.1	1.6%	−2.7	5.6	6.2	3.2%	−3.4	5.7	6.6	3.4%
XNIN	2364	−0.4	2.8	2.8	1.6%	−0.3	3.7	3.8	2.1%	−1.0	3.7	3.9	2.2%
DLHA	2956	0.7	2.3	2.4	1.4%	0.5	2.8	2.9	1.7%	−0.1	2.8	2.8	1.7%
LHAZ	3632	0.9	2.1	2.3	1.5%	−2.0	3.8	4.1	2.6%	−2.3	3.8	4.2	2.7%
**MEAN**	--	−0.3	4.3	4.4	--	1.3	5.1	5.8	--	−0.2	5.1	5.4	--

The EGNOS and UNB3m models have obvious biases in mean values and amplitudes at these stations, particularly at some high altitude locations (e.g., KMIN, XIAG) implying that the EGNOS and UNB3m models cannot describe the seasonal variations of ZTDs very well at these places. This can be explained by the fact that the ENGOS and UNB3m models use regional meteorological data set (The U.S. Standard Atmosphere Supplements) for their constructions, which are of low spatial resolutions (one-dimensional grid tables), therefore the accuracies of these two models in some areas of globe, like China, are degraded [8,9]. In contrast, the IGGtrop model is established based on 3D grids (latitude × longitude × height), and can account for the seasonal and regional variations of the neutral atmosphere behavior much better. Hence, the IGGtrop model shows better performance than the EGNOS and UNB3m models in China [4,5]. 

**Figure 3:**
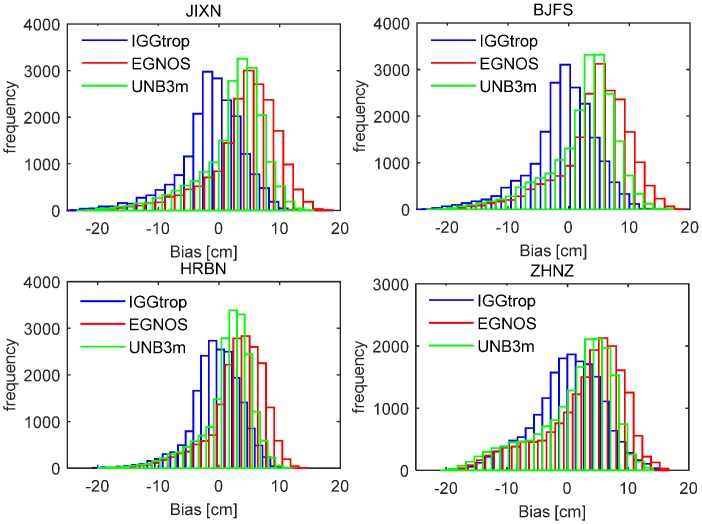
Histogram of the biases for the IGGtrop, EGNOS and UNB3m models over the period from 2009 to 2013 at four exemplary stations of JIXN, BJFS, HRBN and ZHNZ.

To show more detailed distribution of the biases for the IGGtrop, EGNOS and UNB3m models, Figure 3 shows the histograms of biases for these three models over the period from 2009 to 2013 at four exemplary stations. Similar results are obtained at other stations. As can be seen from Figure 3, most of the biases of the IGGtrop model are within ±5 cm and generally follow the standard normal distribution at these four stations. In contrast, most of the biases of the EGNOS and UNB3m models are in the positive axis side with values varying from 3 cm to 10 cm. It further proves that the IGGtrop model has better performance than the EGNOS and UNB3m models in China, and the latter two models are usually show systematic biases in Chinese region. In addition, the histograms of the biases for these three models all show “long tails” in the negative axis side, this is attributed to the extremely high ZTDs appearing in summer which usually cannot be well captured by empirical models (*cf.* Figure 2).

**Figure 4:**
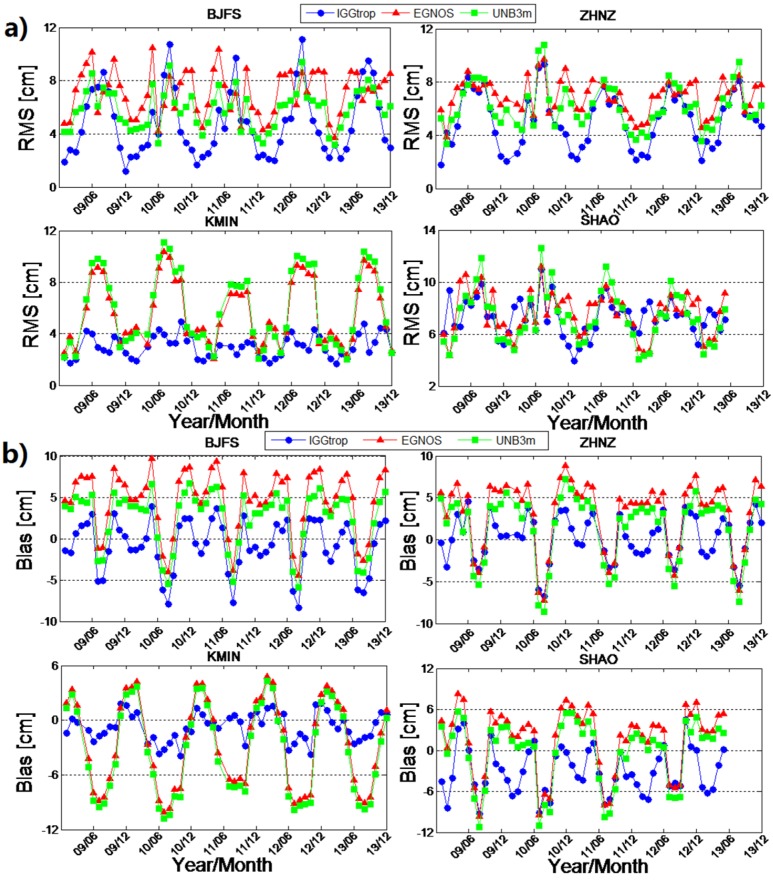
Temporal variations of monthly mean RMS (**a**) and Bias (**b**) for the IGGtrop, EGNOS and UNB3m models at four exemplary stations of BJFS, ZHNZ, KMIN and SHAO.

In order to evaluate the performances of the IGGtrop, EGNOS and UNB3m models in different seasons, we calculate the monthly mean bias and RMS of these three models at 25 stations over five years period from January 2009 to December 2013. The significant seasonal variations are found in both bias (amplitude: 2 cm to 8 cm) and RMS series (amplitude: 2 cm to 5 cm), and the amplitudes and cycles of the variations are station-dependent. Here, we show the results of four exemplary stations in Figure 4.

From Figure 4 we can see that the monthly mean RMS values of these three empirical models (Figure 4a) in summer are usually larger than those in winter. This is due to the larger temporal and spatial variability of the atmospheric water vapor distribution during summer than those in winter, which is the common challenge for empirical tropospheric delay models [21,24]. In addition, the RMS values of the IGGtrop model are generally smaller than those of EGNOS and UNB3m in winter, but in summer, the characteristics are different, for example, at BJFS station, the RMS values of the EGNOS and UNB3m model are smaller than that of the IGGtrop model, but similar performances are obtained for the three models at SHAO station. At high altitude stations, (e.g., KMIN) the IGGtrop model always performs better than the EGNOS and UNB3m models in all seasons. The series of the monthly mean bias (Figure 4b for the IGGtrop model show two peaks in one year at some stations (e.g., BJFS, ZHNZ and SHAO), and the bias values at these stations are generally negative in summer and winter but positive in spring and autumn, which indicating a semiannual variations for the IGGtrop model. 

**Figure 5:**
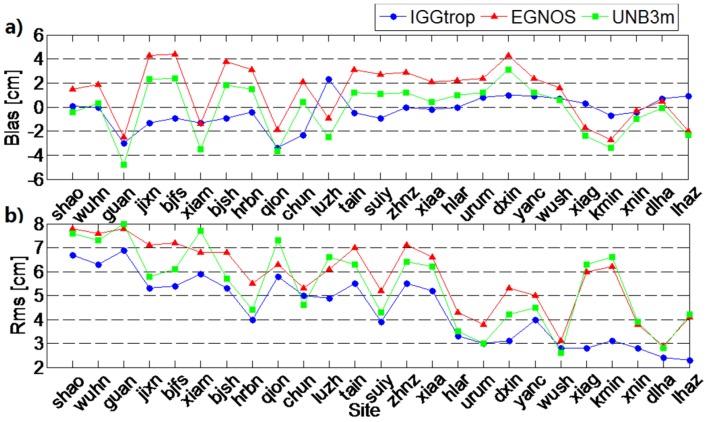
Mean bias (**a**) and RMS (**b**) over the period from 2009 to 2013 for the IGGtrop, EGNOS and UNB3m models. Stations are listed from left to right of the x-axis according to their station height from low to high.

In order to show the performances of the IGGtrop, EGNOS and UNB3m models with respect to station height, we show the RMS and biases of these three models at 25 stations in Figure 5, and the stations are listed according to their heights from low to high. It can be seen that the biases of these three models have no obvious correlation with station height, however, the RMS values decrease with the increasing of heights. The RMS values of IGGtrop (blue) are smaller than those of the EGNOS (red) and UNB3m (green) models at almost all stations. These results, together with the results we have in Table 1, *i.e.,* the STD and relative errors of IGGtrop are generally smaller than those of the EGNOS and UNB3m models in China, we can conclude that the IGGtrop model performs better than the EGNOS and UNB3m models in China, especially at the stations locating at high altitude regions, and these results are consistent with Li *et al.* [4,5] though their analysis focused on a global scale.

### 3.2. Effects of IGGtrop, EGNOS and UNB3m Models on PPP

**Figure 6:**
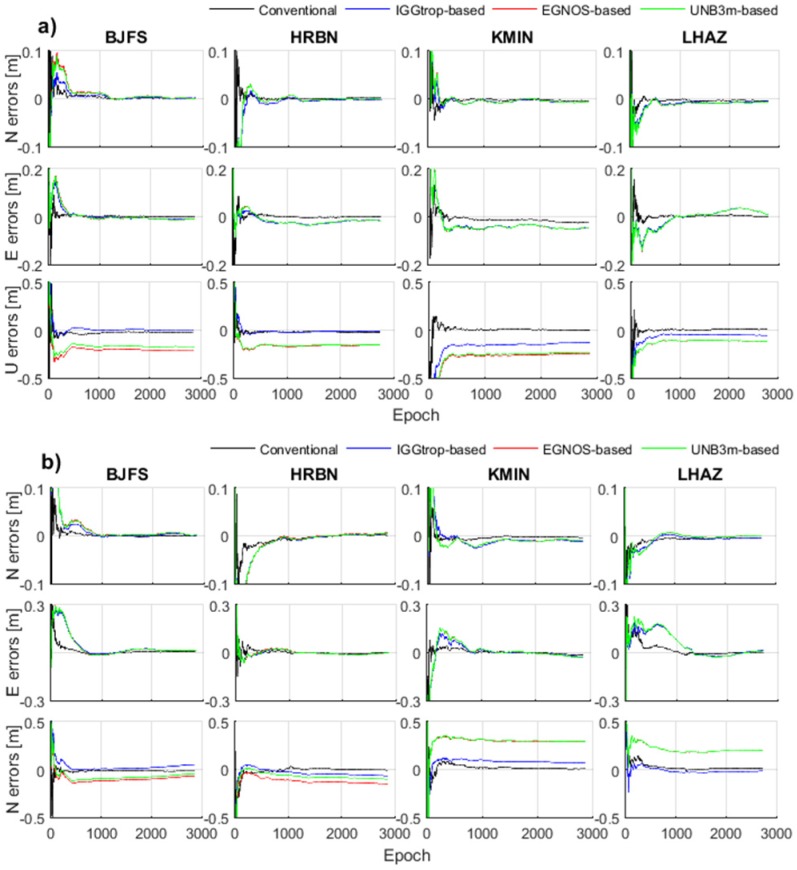
Positioning errors of the conventional PPP and three model-based PPP (IGGtrop-based, EGNOS-based, UNB3m-based) at stations of BJFS, HRBN, KMIN and LHAZ, and the epoch interval is 30 s, the DOY are 28 (**a**) and 200 (**b**) in 2012.

In this section, we implement the IGGtrop, EGNOS and UNB3m models in PPP, and show the positioning accuracies of the PPP solutions based on these three models. Figure 6 shows the positioning errors of the conventional PPP and three modified PPP based on the IGGtrop, EGNOS and UNB3m models (referred to as IGGtrop-based, EGNOS-based and UNB3m-based, respectively) at stations of BJFS, HRBN, KMIN and LHAZ. The day of year (DOY) is 28 for winter (Figure 6a) and is 200 for summer (Figure 6b) in 2012. Comparing the positioning errors of the IGGtrop-based, EGNOS-based and UNB3m-based PPP with those of the conventional PPP, it can be seen that the positioning accuracies as well as convergence time of the PPP solutions are both affected by the errors of ZTD models.

As can be seen from Figure 6, the conventional PPP algorithm has obvious advantages in the convergence time and positioning accuracies compared to the model-based PPP in both horizontal and vertical components. For the three kinds of model-based PPP solutions, the positioning errors in horizontal components are similar, however, significant differences are found in vertical components. The positioning errors in vertical components for the IGGtrop-based PPP are always smaller than those of the EGNOS-based and UNB3m-based PPP at these exemplary stations, and the positioning errors of the EGNOS-based and UNB3m-based PPP solutions are similar, this is attributed to the similar magnitudes of ZTD values calculated by the EGNOS and UNB3m models at these stations (*cf.* Section 3.1).

In addition, for the three kinds of model-based PPP solutions, the convergence time in the horizontal component in summer (Figure 6b) is longer than that in winter (Figure 6a), this is due to the relatively highly variability of the ZTDs in summer. The convergence time of the three model-based PPP solutions are also different, this is caused by varying performances of the ZTD models. Furthermore, we also found that the PPP solutions based on IGGtrop seem to converge faster (taking less time to reach an optimal solution) than those of the EGNOS and UNB3m models implying that the IGGtrop model can accelerate the convergence time compared to the EGNOS and UNB3m models at these exemplary stations.

Table 2 shows the general statistical positioning errors of the conventional PPP solutions and the IGGtrop-based, EGNOS-based and UNB3m-based PPP solutions at 21 selected stations over the whole year period of 2012. It can be seen that the mean positioning errors of the conventional PPP are the smallest among the four kinds of PPP solutions, with 1.0 cm in horizontal component and 1.3 cm in vertical component. The mean positioning error in vertical component for the IGGtrop-based PPP is 15.0 cm which is 30% and 21% smaller than those of the EGNOS-based and UNB3m-based PPP, respectively. This indicates that the IGGtrop model performs better than the EGNOS and UNB3m models in satellite positioning in Chinese region.

**Table 2:** Statistical results of the mean positioning errors in vertical and horizontal components of the conventional PPP, IGGtrop-based, EGNOS-based and UNB3m-based PPP solutions at 21 stations through 2012, respectively.

PPP	Mean Positioning Errors (cm)	
	Horizontal	Vertical
Conventional	1.0	1.3
IGGtrop-based	1.9	15.0
EGNOS-based	2.0	21.4
UNB3m-based	1.9	19.0

**Figure 7:**
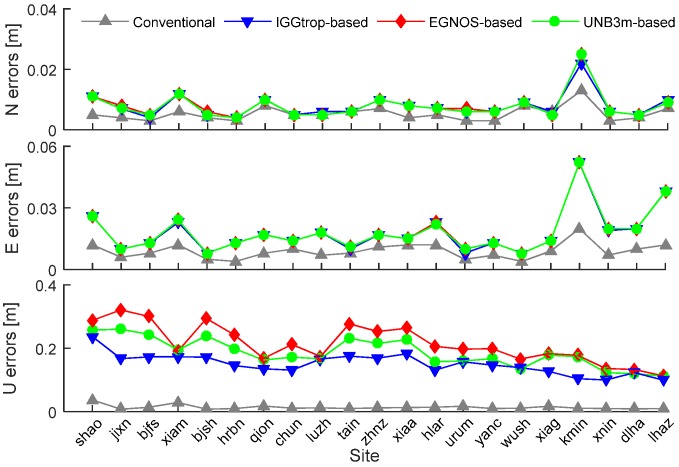
Mean positioning errors of the conventional PPP, IGGtrop-based, EGNOS-based and UNB3m-based PPP solutions over the period from January to December 2012 at selected stations. The upper, medium and bottom panels show the positioning errors in north, east and up directions, respectively.

In order to further analyze the effects of the IGGtrop, EGNOS and UNB3m models on PPP with respect to station height, we plot the annual mean positioning errors of the conventional PPP, IGGtrop-based, EGNOS-based and UNB3m-based PPP solutions at 21 stations in Figure 7. Similar to Figure 5, the stations are listed according to their heights from low to high. It can be seen that the positioning errors of the conventional PPP in both horizontal (North and East) and vertical (Up) components vary little and are of small values at different heights. For the IGGtrop-based, EGNOS-based and UNB3m-based PPP, there are no strong correlations between the horizontal positioning errors and station heights, while the positioning errors in vertical components decrease with the increasing of station heights. This phenomenon is consistent with the variations of model errors with respect to station height (*cf.* Figure 5). In addition, the vertical positioning errors of the IGGtrop-based PPP are smaller than those of the EGNOS-based and UNB3m-based PPP at majority of the selected stations.

**Figure 8:**
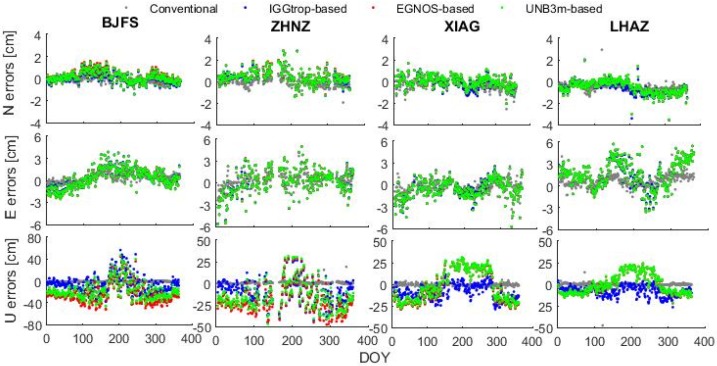
Time series of positioning errors for the conventional, IGGtrop-based, EGNOS-based and UNB3m-based PPP solutions at stations of BJFS, ZHNZ, XIAG and LHAZ, the DOY represents the day of year in 2012.

To illustrate the effects of the IGGtrop, EGNOS and UNB3m models on PPP in different seasons, Figure 8 displays the time series of positioning errors for the conventional, IGGtrop-based, EGNOS-based and UNB3m-based PPP solutions in 2012 with temporal resolution of one-day at four exemplary stations. 

As can be seen from Figure 8, the positioning errors of the conventional PPP (green) in east, north and up components vary little and are of small values throughout the whole year of 2012 (except for a few outliers). However, for the IGGtrop-based (blue), EGNOS-based (red) and UNB3m-based (green) PPP, the positioning errors in north components are relatively stable, but in east components, the errors show slightly seasonal variations with the amplitudes of a few (2–6) centimeters, and the characteristics of variations depend on each specific station, and the reasons deserved to be further studied. Furthermore the errors in east components are usually larger than those in north components which are consistent with the results of Chen *et al.* [24] although the kinematic rather than static PPP mode they used. The vertical positioning errors of the IGGtrop-based, EGNOS-based and UNB3m-based PPP solutions show significant seasonal variations with relatively large amplitudes, and the positioning errors in summer are generally larger than those in winter, which are consistent with the seasonal variations of the models’ errors described in Section 3.1 (*cf.* Figure 4). Furthermore, the amplitudes of the seasonal variations of the vertical positioning errors depend on the specific stations and the tropospheric delay models used in the positioning. From Figure 8 we can also see that the positioning errors in up components for the IGGtrop-based PPP at these four exemplary stations are generally smaller than those of the EGNOS-based and UNB3m-based PPP for most of the time. In summer seasons, the advantage of the IGGtrop-based PPP is usually more obvious at some stations locating at high altitude (e.g., XIAG, LHAZ). However, at BJFS station in summer, the positioning errors in up components for the IGGtrop-based, EGNOS-based and UNB3m-based PPP are similar and more discrete than those in other seasons. This is attributed to the violent variations of atmospheric conditions at BJFS station during this summer, which result in poor accuracies of these empirical models.

In addition, it can be found that the positioning errors in up components for the IGGtrop-based, EGNOS-based and UNB3m-based PPP are always positive in summer at almost all selected stations. This is attributed to the negative biases for the IGGtrop, EGNOS and UNB3m models in summer (*cf.* the model-derived ZTDs in Figure 2 and the vertical positioning errors in Figure 8 at stations of BJFS, ZHNZ and XIAG for comparisons), and the residual ZTD which have not been accounted for by tropospheric delay models would cause positive positioning errors in up components.

## 4. Conclusions

In this paper, performances of the 3D grid-based tropospheric delay model IGGtrop and the widely used models EGNOS and UNB3m under typical Chinese atmospheric conditions are assessed using 5 years’ GPS-derived ZTDs from 25 stations of CMONOC. Furthermore the IGGtrop, EGNOS and UNB3m models are implemented in precise point positioning, and the effects of these three models on positioning are evaluated by processing a whole year’s GPS data from 21 stations of CMONOC. From the study, we found the following:

(1) The RMS of the IGGtrop model is 4.4 cm in the Chinese region, which is about 24% and 19% smaller than that of the EGNOS and UNB3m models, respectively.
(2) The RMS and biases of the IGGtrop, EGNOS and UNB3m models show obviously seasonal variations. In addition, the biases of the IGGtrop model show semiannual variations at some stations.
(3) The mean positioning error of the conventional PPP solutions is about 1.0 cm in horizontal components and is 1.3 cm in vertical components, which is relatively stable throughout the whole year of 2012. The mean vertical positioning error of the PPP solutions based on the IGGtrop model is about 15.0 cm, which is about 30% and 21% smaller than those of the EGNOS and UNB3m models, respectively.
(4) Like the errors of the empirical models, the vertical positioning errors of the PPP solutions obtained by using empirical models also show clear seasonal variations, and the degree of the variations are usually larger than those of the empirical models’ errors.

From such an assessment and the analysis in this paper, we can provide references for the refinement and applications of the IGGtrop, EGNOS and UNB3m models in China. In future, we would carry on our study by evaluating the IGGtrop, EGNOS and UNB3m models in real-time positioning or airborne experiments and conducting extensive work on the developments of the IGGtrop model. 
